# Safety and efficacy of calcitonin gene-related peptide antagonists for cluster headache: a systematic review and meta-analysis

**DOI:** 10.1186/s12883-026-04733-8

**Published:** 2026-02-19

**Authors:** Ro’ya Khanfar, Eithar Radwan, Sama Hattab

**Affiliations:** Department of Medicine, Faculty of Medicine and Allied Medical Sciences, An-Najah National University, Nablus, 44839 Palestine

**Keywords:** Cluster headache, Calcitonin Gene-Related Peptide, CGRP, Galcanezumab

## Abstract

**Background:**

Calcitonin gene-related peptide is an emerging therapeutic target in cluster headache, yet evidence on the preventive efficacy and safety of calcitonin gene-related peptide antagonists remains limited. This systematic review and meta-analysis assessed the efficacy and safety of calcitonin gene-related peptide antagonists in adults with Cluster headache.

**Methods:**

We searched PubMed, Web of Science, Cochrane CENTRAL, ClinicalTrials.gov, and EBSCO for randomized controlled and single-arm trials involving episodic and chronic cluster headache. Safety outcomes evaluate the treatment-emergent adverse events, serious adverse events, and treatment discontinuations, while efficacy outcomes included the change from baseline in the mean number of weekly attacks, the proportion of participants achieving a ≥ 50% or ≥ 30% reduction in weekly attack frequency, and those reporting a “much improved” or “very much improved” status on the Patient Global Impression of Change scale.

**Results:**

Four randomized controlled trials and two single-arm trials were included. Calcitonin gene-related peptide antagonists reduced weekly attacks by a mean of -7.23 (95% CI: − 9.86 to − 4.60). ≥ 50% responder rate was 46% (95% CI: 26%-67%) and ≥ 30% responder rate was 59% (95% CI: 45%-73%). Efficacy effects were larger in episodic than in chronic Cluster headache. The pooled proportion of patients experiencing at least one TEAE was 60% (95% CI: 35%-82%). SAEs (4%, 95% CI: 1%-9%) and discontinuations (3%, 95% CI: 1%-7%) were uncommon.

**Conclusion:**

Calcitonin gene–related peptide antagonists provide preventive benefit with acceptable safety in cluster headache. These findings should be interpreted as supportive but less definitive. Larger, long-term, placebo-controlled randomized trials are needed to confirm efficacy and guide clinical use.

**Supplementary Information:**

The online version contains supplementary material available at 10.1186/s12883-026-04733-8.

## Background

Cluster headache (CH), the most prevalent of the trigeminal autonomic cephalalgias, is defined by attacks of excruciating unilateral orbital, supraorbital, and/or temporal pain, typically lasting between 15 and 180 min when untreated, accompanied by cranial autonomic symptoms [1–3]. The burden of CH extends far beyond physical suffering, imposing a major personal and socioeconomic burden, with productivity reduced by an estimated 65% during active attack periods, especially in the chronic CH population [4–6].

Current management strategies for CH involve acute abortive and preventive therapies [7]. High-flow oxygen and non-oral triptans provide fast relief but are restricted by issues concerning availability and contraindications [8]. Preventive options such as verapamil, lithium, and topiramate are often constrained by a delayed onset of action, monitoring requirements and the need for transitional (bridge) therapy, such as prednisone [9, 10]. However, due to the limited number of rigorous trials, only a few medications have received approval for preventive use [11–13]. Additionally, the precise mechanisms by which these therapies exert their effects are not yet fully understood [14]. As a result, there is ongoing interest in mechanism-based preventive therapies.

The development of targeted therapies has recently focused intensely on Calcitonin Gene-Related Peptide (CGRP), a potent neuropeptide and endogenous vasodilator centrally involved in the pathophysiology of CH [15]. This class of treatment includes monoclonal antibodies targeting the CGRP ligand (e.g., galcanezumab, fremanezumab) or the receptor (e.g., erenumab), as well as small-molecule CGRP receptor antagonists (Gepants) [16]. CH attacks involve activation of the trigeminal-autonomic reflex, leading to the release of CGRP from trigeminal afferent fibers. Elevated CGRP levels observed during attacks, along with the critical finding that exogenous CGRP infusion can trigger cluster attacks, support its central role in trigeminal activation [15, 17].

Despite the proven roles of CGRP antagonist in acute and preventive migraine management, their detailed efficacy and safety data for CH require systematic consolidation since available studies differ in design, populations, dosing, and follow-up, and findings remain inconsistent between episodic and chronic CH. This Systematic Review and single-arm (proportional) Meta-analysis aims to evaluate the safety and efficacy of CGRP antagonists for preventive management of CH by synthesizing data from randomized controlled trials (RCTs) and single-arm trials to inform their potential role in clinical practice.

## Methods

This systematic review and meta-analysis was conducted following the Preferred Reporting Items for Systematic Reviews and Meta-Analysis (PRISMA) [18]. PRISMA checklist is shown in Table S1. It was registered on the International Prospective Register of Systematic Reviews (PROSPERO) with the CRD420251078361 Identification number.

### Search strategy

We searched PubMed, Web of Science, Cochrane CENTRAL, ClinicalTrials.gov, and EBSCO databases to identify relevant studies. The search strategy included the following terms: (erenumab OR fremanezumab OR eptinezumab OR galcanezumab OR atogepant OR ubrogepant OR “CGRP antagonist” OR “Anti-calcitonin Gene-Related Peptide” OR “Calcitonin Gene-Related Peptide Receptor Antagonist”) AND (“cluster headache” OR cluster). The search was limited to English-language publications with no other restrictions, and it included studies published up to July 1, 2025.

### Inclusion/exclusion criteria

We included all clinical trials that met the predefined PICOS criteria: Population (P): adults (≥ 18 years) diagnosed with episodic or chronic CH according to the International Classification of Headache Disorders, 3rd edition (ICHD-3); Intervention (I): calcitonin gene-related peptide (CGRP) antagonists; Comparator (C): placebo or baseline values (for single-arm studies); Outcomes (O): safety outcomes included the incidence of treatment-emergent adverse events (TEAEs), serious adverse events (SAEs), and treatment discontinuations due to adverse events (AEs), while short-term efficacy outcomes included the change from baseline in the mean number of weekly attacks, the proportion of participants achieving a ≥ 50% or ≥ 30% reduction in weekly attack frequency, and those reporting a “much improved” or “very much improved” status on the Patient Global Impression of Change (PGIC) scale; and Study design (S): RCTs and single-arm clinical trials.

Given the limited number of RCTs in CH, single-arm trials were included to enhance evidence synthesis. Any observational study (e.g., cohort, case–control, or cross-sectional studies), reviews, meta-analyses, or case reports were excluded.

### Study selection and data extraction

All identified studies were imported into Rayyan QCRI, an online platform for managing systematic reviews. After removing duplicate studies, two independent reviewers screened the titles and abstracts of the identified studies. Full-text screening of the selected articles was then performed independently by the same reviewers. Any disagreements regarding study eligibility were resolved through discussion. Data extraction was carried out independently by two reviewers using a predesigned Microsoft Excel form, and any differences were addressed and discussed. Extracted data included study characteristics (first author, year of publication, clinicalTrials.gov identifier, study period, study design, country, intervention, cluster type, and sample size) and participant characteristics (age, sex, number of participants in the CGRP antagonist and placebo groups, number of weekly CH attacks at baseline and time since CH diagnosis.

### Risk of bias assessment

Two reviewers independently assessed the risk of bias. For RCTs, the Cochrane Risk of Bias tool (RoB 2.0) was applied, evaluating five domains: randomization process, deviations from intended interventions, missing outcome data, measurement of outcomes, and selection of the reported result. Each domain was rated as low risk, some concerns, or high risk of bias. For single-arm trials, the ROBINS-I V2 tool was used to assess six domains: bias due to confounding, bias in classification of interventions, bias in selection of participants into the study (or into the analysis), bias due to missing data, bias arising from measurement of the outcome and bias in selection of the reported result. Any disagreements between reviewers were resolved through discussion.

### Statistical synthesis

Statistical analyses were performed using Stata 18.0. Proportional meta-analyses were performed, since the reported pooled estimates represented descriptive summaries, not comparative efficacy measures. Dichotomous outcomes were reported in percentages, with 95% confidence intervals (95% CI), while continuous outcomes were expressed as mean difference (MD), with 95% CI. Heterogeneity was quantified using the I^2^ statistic and the chi-square test. Substantial heterogeneity was predefined as an I^2^ value greater than 50% or a *P*-value for the chi-square test of less than 0.05. Der Simonian and Laird random-effects models were used in all analyses. We used the double arcsine method for outcomes with proportions < 20% (e.g., SAEs, discontinuations). *P* values < 0.05 were considered statistically significant. To identify potential sources of heterogeneity and evaluate the robustness of the overall findings, leave-one-out sensitivity analyses and predefined subgroup analyses were conducted. For safety outcomes, subgroup analyses were stratified by study design (RCTs, single-arm studies) and by medication type (eptinezumab, galcanezumab, and erenumab). For efficacy outcomes, subgroup analyses were performed using the same categories, with an additional stratification according to cluster subtype (episodic, chronic). We assessed the publication bias through funnel plots inspection, Egger’s and Begg’s linear regression tests. Due to the small number of included studies (*n* = 6), these formal statistical tests for publication bias were underpowered and may not reliably detect bias. Therefore, results from publication bias assessments should be interpreted with caution, and the possibility of unrecognized reporting bias cannot be excluded. Finally, the level of evidence for the primary outcome was evaluated according to the GRADE guidelines, categorizing the overall quality of evidence as high, moderate, low, or very low [19].

## Results

### Literature searching and baseline characteristics of the selected studies

**Fig. 1:**
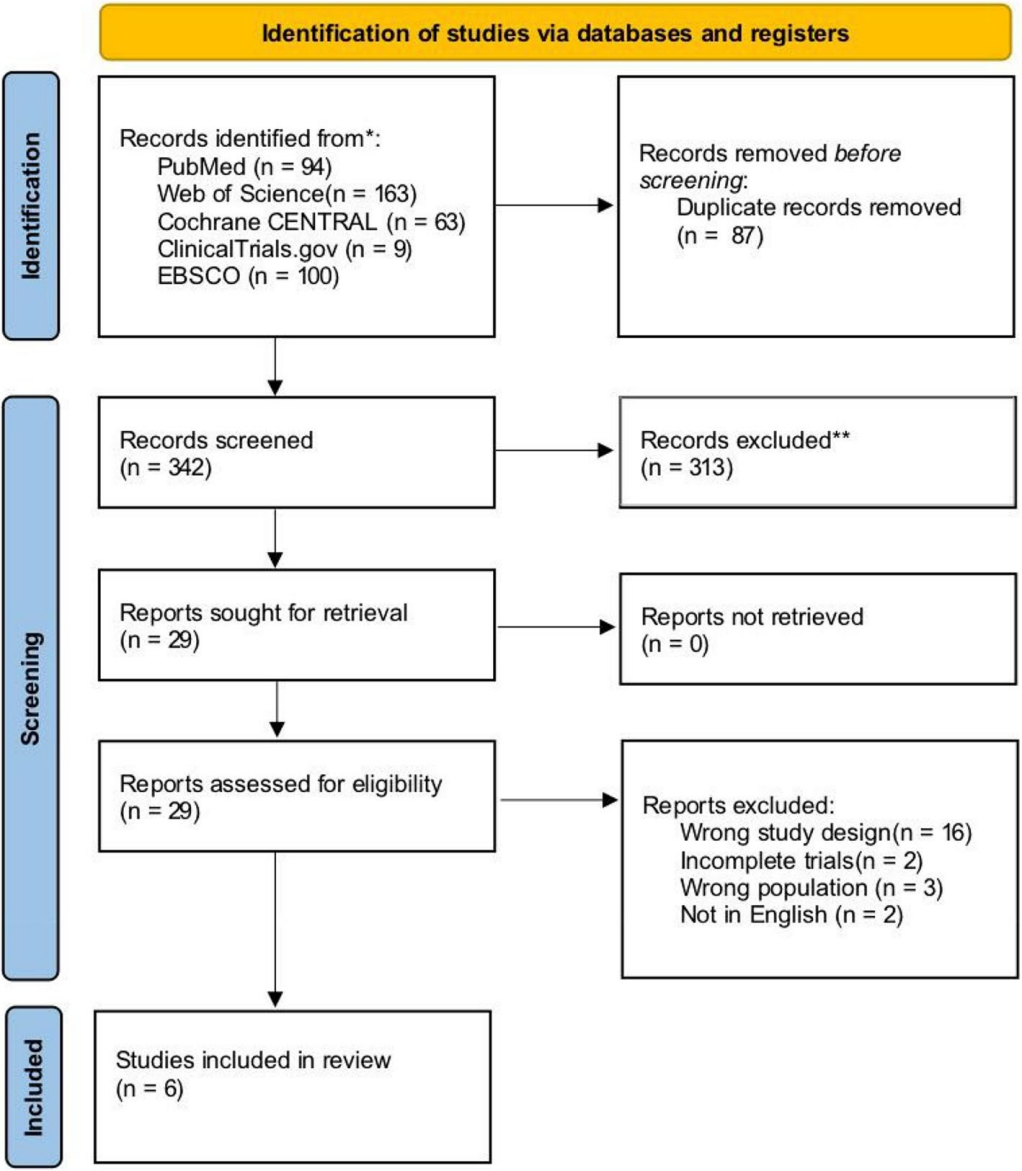
Flow diagram for study inclusion/exclusion

A total of 429 records were identified through database searches: PubMed (*n* = 94), Web of Science (*n* = 163), Cochrane CENTRAL (*n* = 63), ClinicalTrials.gov (*n* = 9), and EBSCO (*n* = 100). After removing 87 duplicates, 342 unique records were screened based on title and abstract. Of these, 313 were excluded, leaving 29 articles for full-text assessment. Following the eligibility evaluation, 23 studies were excluded, and six studies ultimately fulfilled the inclusion criteria and were incorporated into the review [20–25]. A detailed flowchart of the study selection process is shown in Fig. 1.

The literature search included four double-blind, RCTs and two single-arm open-label clinical trials, published between 2019 and 2025, conducted across Europe, North America, and Asia, with follow-up durations ranging from several weeks to months. One of the included single-arm trials [23], is an extension of the RCT [21], so we didn’t include it in the same quantitative synthesis to avoid double counting. These studies enrolled 1,019 patients with CH. Among them, 788 patients received CGRP monoclonal antibodies, eptinezumab, galcanezumab, or erenumab, while 231 received a placebo in the RCTs. Both episodic and chronic CH populations were represented. The key characteristics of the included studies are presented in Table S3.

Across studies, the mean age of participants ranged from 44.2 to 48.3 years, with a predominance of male patients (approximately 65–75%). The mean number of weekly CH attacks at baseline varied between 15.2 and 21.2 attacks per week, and the average disease duration ranged from 8.4 to 17.6 years. Baseline characteristics were well balanced between intervention and placebo groups in the RCTs. Baseline demographics and clinical features are detailed in Table 1.

**Table 1:** Baseline demographics and clinical features of participants

ID	CGRP group				Placebo (for RCTs)			
	Age mean ± std	Male/female	number of weekly CH attacks at baseline	Time since CH diagnosis	Age mean ± std	Male/female	number of weekly CH attacks at baseline	Time since CH diagnosis
Jensen RH et al. (2025) [25]	44.2 ± 11.1	85/27	15.2 ± 8.1	10.5 ± 8.6	43.9 ± 11	93/24	15.7 ± 8.3	9.5 ± 8.5
Tassorelli, Cristina et al. (2025) [20]	45.2 ± 10.8	84/47	17.9 ± 16.7	7.3 ± 5.8	NA	NA	NA	NA
Láinez et al. (2022) [23]	44.9 ± 10.9	169/36	18.9 ± 10.2	8.0 ± 7.1	NA	NA	NA	NA
Mecklenburg et al. (2025) [24]	48.3 ± 10.7	30/11	21.2 ± 9.0	7.6 ± 7.0	49.6 ± 10.3	30/10	21.7 ± 10.6	9.0 ± 7.3
Goadsby et al. (2019) [22]	47 ± 11	41/8	17.8 ± 10.1	15.8 ± 11.1	45 ± 11	47/10	17.3 ± 10.0	17.6 ± 11.5
Dodick et al. (2020) [21]	45.6 ± 11.0	86/31	19.2 ± 9.8	7.7 ± 6.6	44.4 ± 10.8	86/34	18.5 ± 10.7	8.4 ± 7.5

*CH* Cluster headache, *RCT* Randomized controlled trial

### Risk of bias assessment

**Fig. 2:**
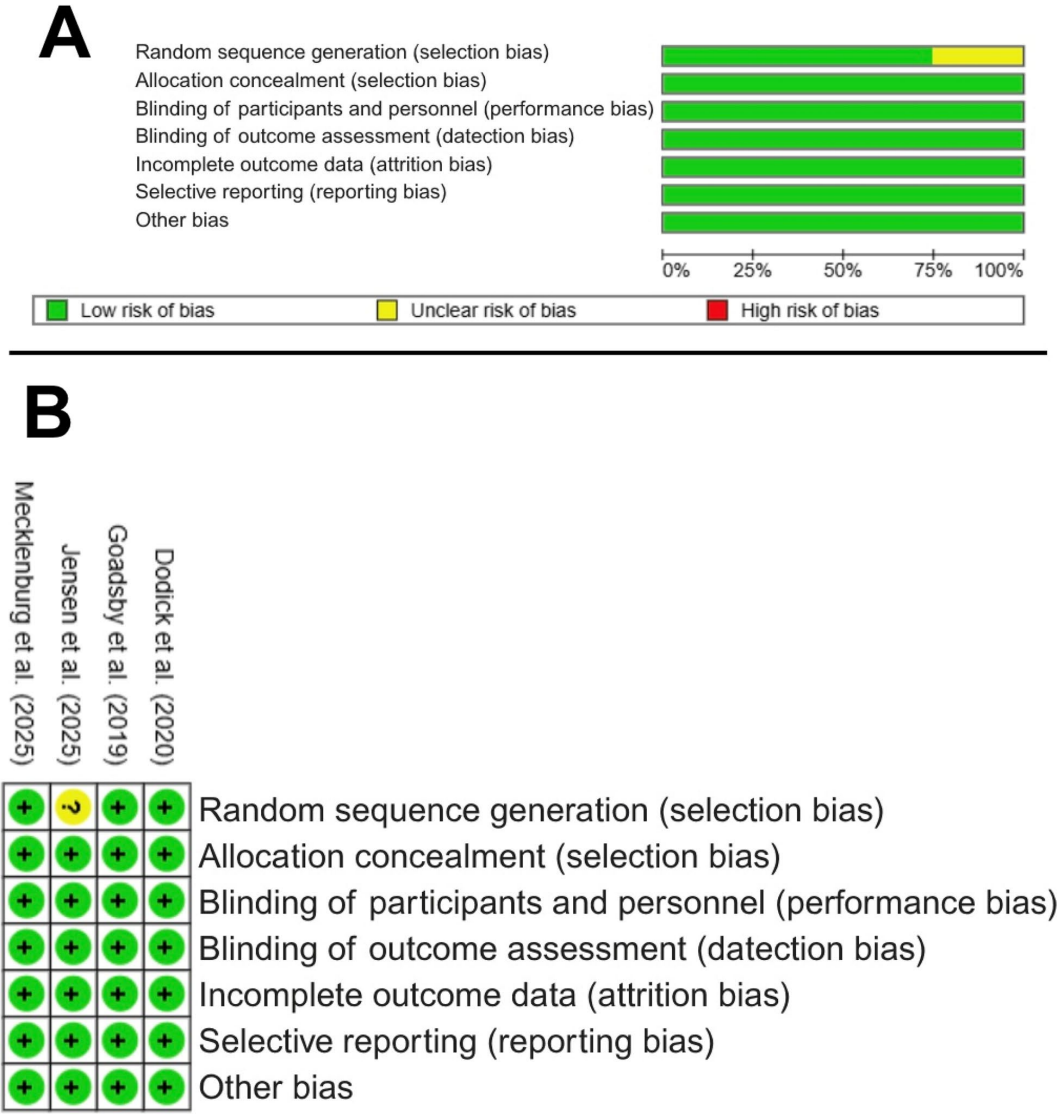
Cochrane risk of bias assessment. **A** The risk of bias graph. **B** The risk of bias summary

Risk of bias assessment is presented in Fig. 2 for RCTs and Table S4 for single-arm trials. The majority of RCTs were judged to be at low risk of bias, with adequate randomization and blinding procedures. The two single-arm studies were rated at moderate risk of bias, primarily due to the absence of a comparator group and potential confounding.

### Main safety outcomes

**Fig. 3:**
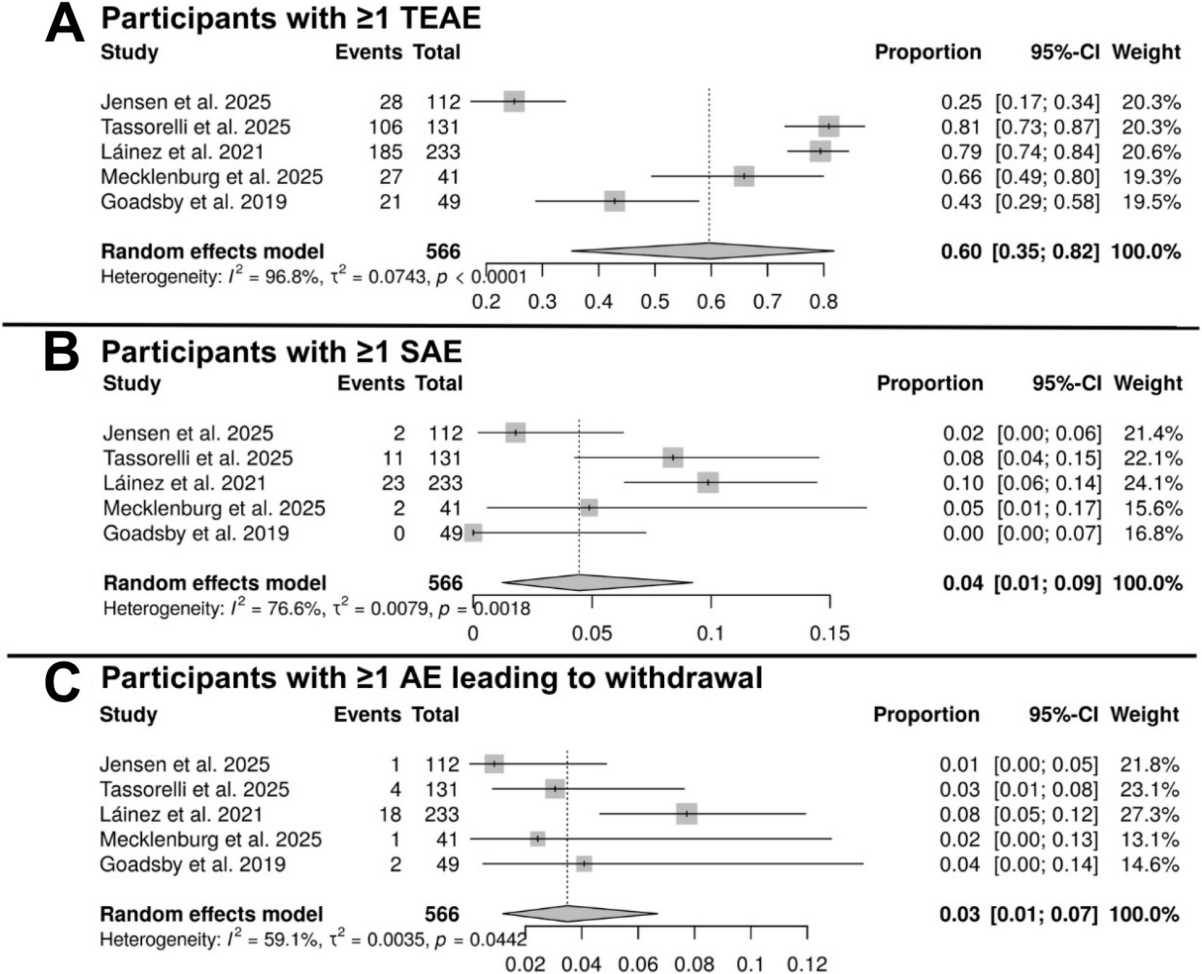
Pooled safety outcomes of CGRP antagonists in cluster headache. **A** Proportion of participants experiencing at least one treatment-emergent adverse event (TEAE). **B** Proportion of participants experiencing at least one serious adverse event (SAE). **C** Proportion of participants with an adverse event leading to treatment withdrawal. CI: confidence interval, TEAEs: Treatment-Emergent Adverse Events, SAEs: Serious Adverse Events, AEs: Adverse Events

A total of 5 studies comprising 566 participants were included in the pooled safety analysis (Fig. 3). All pooled results represent observed within-group outcomes and do not constitute placebo-controlled treatment effect estimates. The pooled proportion of patients experiencing at least one TEAE was 60% (95% CI: 35%—82%), indicating that approximately two-thirds of treated patients reported at least one TEAE (Fig. 3A). SAEs were infrequent, with a pooled proportion of 4% (95% CI: 1%−9%). Treatment discontinuation due to AEs occurred in a minority of patients, with a pooled rate of 3% (95% CI: 1%−7%). All analyses demonstrated substantial heterogeneity with I^2^ ranging from 59.1% to 96.8%.

Subgroup analysis (Table 2, Figure S1 and S2) by study design, RCTs reported lower TEAE proportions (44%, 95% CI: 21%−68%) compared with single-arm trials (80%, 95% CI: 76%−84%). In addition, RCTs represented a lower SAEs and discontinuation rates but remained low in both groups. With respect to individual medications, the pooled TEAE proportions were 54% (95% CI: 5%−97%) for eptinezumab, 63% (95% CI: 26%−93%) for galcanezumab, and 60% (95% CI: 35%−82%) for erenumab. The corresponding pooled SAE proportions were 5% (95% CI: 0%−13%), 4% (95% CI: 0%−19%), and 5% (95% CI: 1%−17%), respectively. Discontinuation rates remained low across all agents, at 2% (95% CI: 0%−5%) for eptinezumab, 7% (95% CI: 4%−10%) for galcanezumab, and 3% (95% CI: 1%−7%) for erenumab.

**Table 2:** Subgroup analyses for safety outcomes

Subgroup		TEAEs				SAEs				Discontinuation rate			
		No	ES (95% CI)	P	I^2^	No	ES (95% CI)	P	I^2^	No	ES (95% CI)	P	I^2^
Study design	RCT	3	0.44 (0.21–0.68)	*p* < 0.0001	90.90%	3	0.01 (0.00–0.05)	0.2535	27.1%,	3	0.02 (0.00–0.04)	0.3884	0.00%
	Single-arm clinical trial	2	0.80 (0.76–0.84)	0.7466	0.00%	2	0.09 (0.06–0.13)	0.6746	0.00%	2	0.05 (0.02–0.11)	0.0442	59.10%
Medication	Eptinezumab	2	0.54 (0.05–0.97)	*p* < 0.0001	98.80%	2	0.05 (0.00–0.13)	0.0187	81.90%	2	0.02 (0.00–0.05)	0.2648	19.60%
	Galcanezumab	2	0.63 (0.26–0.93)	*p* < 0.0001	95.8%,	2	0.04 (0.00–0.19)	0.0013	90.30%	2	0.07 (0.04–0.10)	0.4387	0.00%
	Erenumab	1	0.60 (0.35–0.82)	N/A	N/A	1	0.05 (0.01–0.17)	N/A	N/A	1	0.03 (0.01–0.07)	N/A	N/A

*TEAEs* Treatment-Emergent Adverse Events, *SAEs* Serious Adverse Events

A total of 14 adverse events (Table 3) were analyzed across the included studies. The most commonly reported events were injection site reactions (13%, 95% CI: 2%−31%; I^2^ = 90.7%) and injection site pain (13%, 95% CI: 8%−18%; I^2^ = 12.8%). Other frequent adverse events included nasopharyngitis (8%, 95% CI: 2%−17%; I^2^ = 88.7%), fatigue (7%, 95% CI: 2%−14%; I^2^ = 83.1%), and COVID-19 infection (9%, 95% CI: 0%−39%; I^2^ = 97.1%). Less common events were constipation (5%, 95% CI: 3%−7%; I^2^ = 0%), nausea (5%, 95% CI: 3%−7%; I^2^ = 0%), pruritus (5%, 95% CI: 1%−10%; I^2^ = 58.8%), and dizziness (4%, 95% CI: 1%−7%; I^2^ = 46.8%). Rare events included vomiting (3%, 95% CI: 0%−6%; I^2^ = 44.3%), pyrexia (3%, 95% CI: 1%−6%; I^2^ = 0.0%), influenza (6%, 95% CI: 0%−17%; I^2^ = 93.5%), back pain (12%, 95% CI: 7%−18%; I^2^ = 42.7%), and arthralgia (3%, 95% CI: 1%−5%; I^2^ = 43.3%). No death cases were reported. Considerable heterogeneity (I^2^ > 50%) was observed in several outcomes, notably fatigue, nasopharyngitis, injection site reactions, and back pain, suggesting variability among study estimates.

**Table 3:** Pooled analysis of individual adverse events

Adverse event	No. of included studies	Rate (95% CI)	I^2^
Constipation	4	5% (3%−7%)	0.00%
Fatigue	4	7% (2%−14%)	83.10%
Nasopharyngitis	5	8% (2%−17%)	88.70%
COVID-19 infection	2	9% (0%−39%)	97.10%
Death	5	0% (0%−0%)	0.00%
Nausea	3	5% (3%−7%)	0.00%
Vomiting	2	3% (0%−6%)	44.30%
Back pain	2	12% (7%−18%)	42.70%
Influenza	3	6% (0%−17%)	93.50%
Dizziness	3	4% (1%−7%)	46.80%
Arthralgia	2	3% (1%−5%)	43.30%
Injection site reactions	3	13% (2%−31%)	90.70%
Injection site pain	2	13% (8%−18%)	12.80%
Injection site pruritus	2	6% (4%−9%)	0.00%
Pruritus	2	5% (1%−10%)	58.80%
Pyrexia	2	3% (1%−6%)	0.00%

### Main short-term efficacy outcomes

**Fig. 4:**
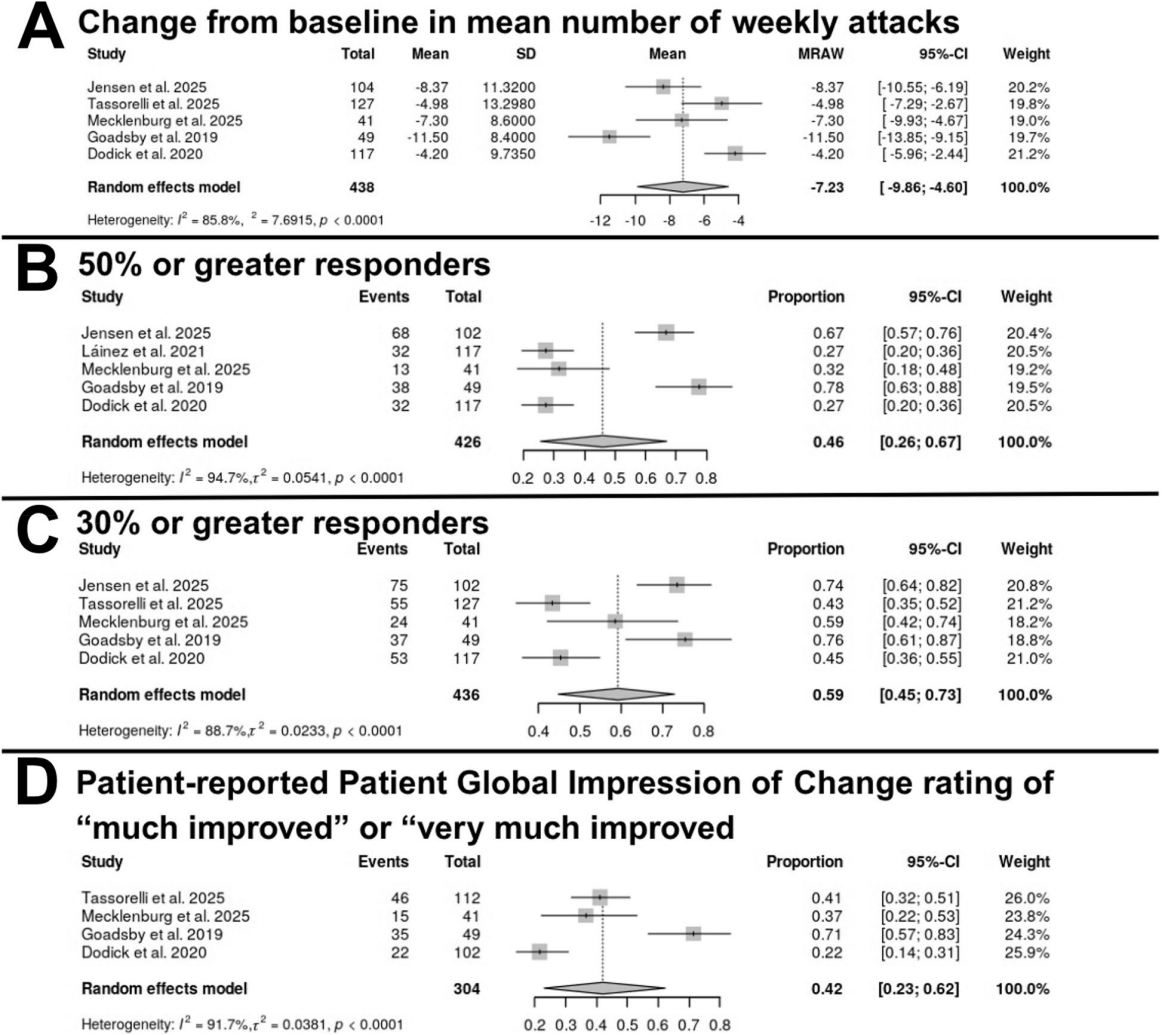
Pooled efficacy outcomes of CGRP antagonists in cluster headache. **A** Mean change from baseline in the weekly frequency of attacks. **B** Proportion of participants achieving at least a 50% reduction in attack frequency. **C** Proportion of participants achieving at least a 30% reduction in attack frequency. **D** Proportion of participants reporting “much improved” or “very much improved” on the Patient Global Impression of Change

Efficacy outcomes were extracted at the earliest common post-baseline time point, which ranged from the 4th week to the 6th week according to the study, since most trials included efficacy outcomes in the 4th week, with one study [24] reporting outcomes at weeks 5/6. These time points were pooled because they represented the primary assessment window in all trials. Across the included studies, the pooled efficacy outcomes (Fig. 4) indicated a reduction in the mean number of weekly attacks, with a mean change of −7.23 (95% CI: −9.86 to −4.60). Further analyses of patient-reported outcomes revealed a pooled proportion of 46% (95% CI: 26%−67%) of patients achieving a ≥ 50% reduction in attacks and 59% (95% CI: 45%−73%) achieving a ≥ 30% reduction. The pooled proportion of patients reporting a “much improved” or “very much improved” status on the PGIC scale was 42% (95% CI: 23%−62%). Notably, all analyses demonstrated substantial heterogeneity, with I^2^ values ranging from 84.7% to 91.7% (all *p* < 0.0001).

Subgroup analyses were conducted to explore potential sources of heterogeneity based on study design, medication, and cluster type (Table S5, Figure S3-S5). The mean reduction in weekly attacks was greater in RCTs (−7.79, 95% CI: −10.97 to −4.61) compared to single-arm trials (−4.98, 95% CI: −7.29 to −2.67). When comparing medications, galcanezumab showed the largest pooled mean reduction of −7.8 (95% CI: −14.96 to −0.65), although this was based on only two studies with high heterogeneity. It also stayed the most effective medication according to other efficacy outcomes. Analysis by cluster type revealed a greater mean reduction of −9.90 (95% CI: −12.97 to −6.84) in patients with episodic conditions versus −7.23 (95% CI: −9.86 to −4.60) in those with chronic conditions. For responder rates, RCTs showed the pooled proportion of patients achieving a ≥ 50% reduction in weekly attacks was 0.51 (95% CI: 0.26–0.76), while the proportion achieving a ≥ 30% reduction was 0.63 (95% CI: 0.47–0.78). For patient-reported global improvement, 42% (95% CI: 0.15–0.73) of patients were rated as “much improved” or “very much improved” on the PGIC scale. Interestingly, the pooled proportion of patients with a ≥ 30% reduction in the episodic subgroup was 74% (95% CI: 67%−81%, I^2^ = 0%), while in the chronic subgroup it was 47% (95% CI: 40%−54%, I^2^ = 0%). Similarly, the pooled proportion of ≥ 50% responders in the episodic subgroup was 71% (95% CI: 60%−81%, I^2^ = 45.4%) and ≥ 50% responders in the chronic subgroup was 30% (95% CI: 25%−35%, I^2^ = 0%).

### Publication bias and GRADE assessment

The publication bias of the pooled TEAE (Egger’s test: *P* = 0.578; Begg’s test: *P* = 0.462), pooled ≥ 50% responder rate (Egger’s test: *P* = 0.765; Begg’s test: *P* = 0.2207) and pooled ≥ 30% responder rate (Egger’s test: *P* = 0.673; Begg’s test: *P* = 1.000) were examined with both Egger’s regression test and Begg’s rank correlation test and they were non-significant. A wide but symmetrical distribution of studies was observed in the funnel plot, indicating no statistical evidence of publication bias (Figure S6). However, the reliability of these results may be limited by the small number of included studies (*n* = 6) [26]. Certainty of evidence was assessed using the GRADE approach for only RCT outcomes. The certainty of evidence was was rated as Moderate for patients with at least TEAE, change from baseline in weekly attacks, ≥ 30% responder rate, and ≥ 50% responder rate, and Low for the outcomes; patients with at least SAE, discontinuations due to AEs and proportion of patients reporting a “much improved” or “very much improved” status on the PGIC scale. A detailed summary of all judgments is presented in Table S6. However, Evidence from single-arm trials, which lack a comparator, was presented separately as very low-certainty descriptive evidence.

### Sensitivity analysis

The robustness of the summary results was determined through a sensitivity analysis, where each study was systematically eliminated. The findings indicated that none of the summary results, nor their 95% CIs, were significantly affected by the exclusion of any single study. Notably, Exclusion of the [24] study did not materially alter the pooled effect sizes or statistical significance for attack frequency reduction and responder rates, indicating that inclusion of the slightly later assessment did not bias the overall short-term efficacy conclusions. This suggests generally robust and reliable results from our meta-analysis, as shown in Figures S7 and S8.

## Discussion

This systematic review and meta-analysis evaluated the safety and efficacy of CGRP antagonists in CH across six clinical trials. Overall, CGRP antagonists demonstrated clinically relevant reductions in attack frequency and responder rates, with an acceptable safety profile.

Substantial heterogeneity was observed across almost all pooled analyses. This is likely driven by differences in study design (RCTs vs. single-arm trials), follow-up duration, dosing regimens, and the proportions of episodic versus chronic CH participants. Larger short-term improvements were observed in episodic CH compared with chronic CH. Although the biological distinctions between episodic and chronic CH remain incompletely understood [14], available evidence suggests that episodic CH may involve more transient CGRP-driven activation of the trigeminal–autonomic reflex. In contrast, chronic CH may be sustained by additional central sensitization mechanisms less responsive to CGRP blockade [15, 16]. These differences may partly explain the comparatively smaller treatment effects observed in chronic CH, but further research is needed to clarify these relationships. However, these subgroup findings are still methodologically limited. They should be considered hypothesis-generating rather than confirmatory, since there remains a risk of early resolution during the episodic CH phase, although episodic headache trials attempt to mitigate this by enrolling participants early in the episodic period.

Consistent with these findings, multiple studies have highlighted the preventive value of CGRP inhibition in CH and results differ between these studies according to the used medication, CH type and study design. A systematic review and meta-analysis by Barbosa da Silva et al. showed that galcanezumab reduced weekly CH attacks by at least 50% in 76% of cases, with 79% of patients reporting clinical improvement, demonstrating both efficacy and good tolerability [27]. Similarly, Andrews et al. demonstrated that galcanezumab significantly reduces the total pain burden in patients with episodic CH, encompassing not just attack frequency but also duration and severity [28]. A recent study by Hong et al. further supported these results, reporting that galcanezumab reduced daily CH attacks by ≥ 50% in 86% of patients during the first episode and 64% in a subsequent episode, again with good tolerability [29]. Real-world case series by Silvestro et al. Showed that monthly administration of erenumab effectively reduced both the frequency and intensity of CH attacks, particularly in patients who have not responded to or tolerated traditional therapies [30]. Additionally, Serrão et al. suggested that atogepant may provide preventive benefit in refractory CH, with meaningful reductions in attack frequency without serious adverse effects [31]. On the other hand, fremanezumab failed to demonstrate significant efficacy in both episodic and chronic CH, leading to early termination of its clinical trials [32]. Several of these prior studies have reported favorable outcomes with CGRP antagonists in episodic CH. However, these findings are predominantly derived from short-term or non-comparative analyses and the current evidence base does not permit definitive conclusions regarding differential efficacy between episodic and chronic CH. Collectively, these data reinforce that CGRP antagonists may represent a promising option for preventive therapy, especially for refractory CH.

Safety analyses across included trials indicate that CGRP antagonists are well-tolerated. Approximately two-thirds of patients experienced at least one TEAE. Serious adverse events and treatment discontinuations were rare. Importantly, no significant cardiovascular or hepatic toxicities were reported, consistent with findings from migraine trials [33, 34]. The absence of vasoconstrictive properties makes CGRP antagonists particularly advantageous for patients with cardiovascular risk, which is an important consideration since it predominantly affects middle-aged men with higher rates of smoking and comorbidities. This represents a substantial clinical benefit compared with traditional vasoconstrictive agents such as triptans [35].

To our knowledge, this is the first systematic review and meta-analysis to synthesize evidence across all CGRP antagonists as a therapeutic group in CH. However, several limitations should be acknowledged. First, the number of available trials is small, which limits statistical power and generalizability. Second, substantial heterogeneity was observed, likely reflecting differences in cluster subtype, dosing intervals, follow-up duration, and the non-uniform timing of outcome assessments across studies. Third, the inclusion of single-arm studies introduces additional uncertainty due to the absence of comparator groups. Fourth, most studies reported only short- to medium-term outcomes, and long-term efficacy and safety, particularly in chronic CH, remain insufficiently characterized. Fifth, limited reporting of potential effect modifiers such as smoking status, sex, or comorbidities prevented more detailed subgroup analyses. Sixth, this review focused exclusively on CGRP monoclonal antibodies, as no eligible trials were available for small-molecule CGRP receptor antagonists (gepants). Seventh, although episodic CH trials applied eligibility criteria to reduce early spontaneous remission, heterogeneity in enrollment timing, baseline disease phase and outcome assessment across studies still limits confidence in pooled efficacy estimates. Finally, most participants were adults aged 18–60 years; therefore, extrapolation to pediatric or older populations should be made with caution.

This systematic review suggests that CGRP monoclonal antibodies may offer a promising preventive option for both episodic and chronic CH, although the evidence remains limited, particularly for chronic CH. These findings highlight the potential relevance CH subtype when interpreting available evidence. However, given current methodological limitations, treatment decisions should remain individualized and guided by clinical judgment until more robust, placebo-controlled comparative data become available. Clinically, CGRP monoclonal antibodies may be considered as part of the preventive treatment algorithm, either alone or in combination with established agents such as verapamil, while acknowledging the need for further high-quality trials.

Future research should focus on conducting more Placebo-controlled RCTs and head-to-head comparisons between different CGRP antagonists to identify the most effective agent. Long-term follow-up studies are needed to assess sustained efficacy, safety, quality of life, disability measures and potential immunogenicity. Finally, combination therapy trials integrating CGRP antagonists with conventional preventive treatments may offer improved outcomes for patients with refractory chronic CH.

## Conclusion

This systematic review and meta-analysis provides evidence supporting the preventive use of CGRP monoclonal antibodies in CH. CGRP antagonists demonstrated clinically meaningful reductions in weekly attack frequency and responder rates, with an overall favorable safety profile. Larger short-term improvements were observed in episodic disease. However, these findings should be considered hypothesis-generating rather than confirmatory and require validation in adequately powered, placebo-controlled randomized trials. Adverse events were generally mild, and serious adverse events or treatment discontinuations were rare, highlighting the tolerability of these agents.

Despite the encouraging results, the current evidence is limited by the small number of trials, substantial heterogeneity, short- to medium-term follow-up, and the inclusion of single-arm studies. Therefore, while CGRP monoclonal antibodies represent a promising preventive option, especially for refractory cases, caution is warranted in interpreting these findings for both episodic and chronic CH populations.

Future high-quality, placebo-controlled, and head-to-head trials with longer follow-up are essential to confirm long-term efficacy and safety, clarify differential effects between cluster subtypes, and optimize therapeutic strategies. Integration of CGRP antagonists with conventional preventive therapies may offer additional benefit for patients who remain refractory to current standard treatments.

## Supplementary Information

Supplementary Material 1.

## Data Availability

All data analyzed in this study were included in the manuscript or as supplementary materials. The datasets used in the analysis or entered into statistical software can be obtained from the corresponding author upon making a reasonable request.

## Abbreviations

AEs: Adverse Events
CGRP: Calcitonin Gene-Related Peptide
CH: Cluster Headache
CI: Confidence Interval
GRADE: Grading of Recommendations, Assessment, Development and Evaluation
ICHD-3: International Classification of Headache Disorders, 3rd Edition
MD: Mean Difference
PGIC: Patient Global Impression of Change
PICOS: Population, Intervention, Comparator, Outcomes, Study design
PRISMA: Preferred Reporting Items for Systematic Reviews and Meta-Analyses
RCT: Randomized Controlled Trial
ROBINS-I: Risk of Bias in Non-Randomized Studies of Interventions
RoB 2.0: Cochrane Risk of Bias Tool, Version 2
SAEs: Serious Adverse Events
Std: Standard Deviation
TEAEs: Treatment-Emergent Adverse Events
