# Supplementary material for: Safety and efficacy of calcitonin gene-related peptide antagonists for cluster headache: a systematic review and meta-analysis
Source: BMC Neurol. 2026 Feb 19;26:195. doi: 10.1186/s12883-026-04733-8 (PMC13019765; doi:10.1186/s12883-026-04733-8)

Supplementary material

Safety and efficacy of Calcitonin gene-related peptide antagonist for Cluster headache: A Systematic Review and Meta-analysis

Contents

Table S1. PRISMA 2020 Checklist 3

Table S2. PRISMA Abstract Checklist 7

Table S3. Key characteristics of the included studies 8

Table S4. Risk of bias summary for non-randomized studies (ROBINS-I) tool for single-arm trials 10

Table S5. Subgroup analyses for efficacy outcomes 11

Table S6. GRADE summary of findings for randomized controlled trials (proportional outcomes) 12

Figure S1. Subgroup Analysis of Safety Outcomes by Study Design; (A) Participants with ≥1 TEAE, (B) Participants with ≥1 SAE, (C) Participants with ≥1 AE leading to withdrawal 15

Figure S2. Subgroup Analysis of Safety Outcomes by Medication Type;(A) Participants with ≥1 TEAE, (B) Participants with ≥1 SAE, (C) Participants with ≥1 AE leading to withdrawal 15

Figure S3. Subgroup analysis of efficacy outcomes by study design; (A) change from baseline in number of weekly Attacks, (B) 50% or greater responders, (C) 30% or greater responders, (D) Patient-reported Patient Global Impression of Change rating of "much improved" or "very much improved" 16

Figure S4. Subgroup analysis of efficacy outcomes by medication type; (A) change from baseline in number of weekly Attacks, (B) 50% or greater responders, (C) 30% or greater responders, (D) Patient-reported Patient Global Impression of Change rating of "much improved" or "very much improved" 16

Figure S5. Subgroup analysis of efficacy outcomes Cluster headache type (Episodic vs Chronic); (A) change from baseline in number of weekly Attacks, (B) 50% or greater responders, (C) 30% or greater responders, (D) Patient-reported Patient Global Impression of Change rating of "much improved" or "very much improved" 17

Figure S6. Funnel Plots for Publication Bias for the outcomes: (A) Participants with ≥1 TEAE, (B) 50% or greater responders, (C) 30% or greater responders 18

Figure S7. Leave-One-Out Sensitivity Analysis for Safety Outcomes; (A) Participants with ≥1 TEAE, (B) Participants with ≥1 SAE, (C)Participants with ≥1 AE leading to withdrawal 19

Figure S8. Leave-One-Out Sensitivity Analysis for Efficacy Outcomes; (A) 50% or greater responders, (B) 30% or greater responders, (C) Patient-reported Patient Global Impression of Change rating of "much improved" or "very much improved" 19

Table S1. PRISMA 2020 Checklist

Section and Topic	Item #	Checklist item	Location where item is reported
**TITLE**			
Title	1	Identify the report as a systematic review.	page 1
**ABSTRACT**			
Abstract	2	See the PRISMA 2020 for Abstracts checklist.	Table S2
**INTRODUCTION**			
Rationale	3	Describe the rationale for the review in the context of existing knowledge.	Page 3
Objectives	4	Provide an explicit statement of the objective(s) or question(s) the review addresses.	Page 3
**METHODS**			
Eligibility criteria	5	Specify the inclusion and exclusion criteria for the review and how studies were grouped for the syntheses.	Page 4
Information sources	6	Specify all databases, registers, websites, organisations, reference lists and other sources searched or consulted to identify studies. Specify the date when each source was last searched or consulted.	Page 3
Search strategy	7	Present the full search strategies for all databases, registers and websites, including any filters and limits used.	Page 3 and 4
Selection process	8	Specify the methods used to decide whether a study met the inclusion criteria of the review, including how many reviewers screened each record and each report retrieved, whether they worked independently, and if applicable, details of automation tools used in the process.	Page 4
Data collection process	9	Specify the methods used to collect data from reports, including how many reviewers collected data from each report, whether they worked independently, any processes for obtaining or confirming data from study investigators, and if applicable, details of automation tools used in the process.	Page 4
Data items	10a	List and define all outcomes for which data were sought. Specify whether all results that were compatible with each outcome domain in each study were sought (e.g. for all measures, time points, analyses), and if not, the methods used to decide which	Page 4

Section and Topic	Item #	Checklist item	Location where item is reported
		results to collect.	
	10b	List and define all other variables for which data were sought (e.g. participant and intervention characteristics, funding sources). Describe any assumptions made about any missing or unclear information.	Page 4
Study risk of bias assessment	11	Specify the methods used to assess risk of bias in the included studies, including details of the tool(s) used, how many reviewers assessed each study and whether they worked independently, and if applicable, details of automation tools used in the process.	Page 4
Effect measures	12	Specify for each outcome the effect measure(s) (e.g. risk ratio, mean difference) used in the synthesis or presentation of results.	Page 4 and 5
Synthesis methods	13a	Describe the processes used to decide which studies were eligible for each synthesis (e.g. tabulating the study intervention characteristics and comparing against the planned groups for each synthesis (item #5)).	NA
	13b	Describe any methods required to prepare the data for presentation or synthesis, such as handling of missing summary statistics, or data conversions.	Page 4
	13c	Describe any methods used to tabulate or visually display results of individual studies and syntheses.	Page 4 and 5
	13d	Describe any methods used to synthesize results and provide a rationale for the choice(s). If meta-analysis was performed, describe the model(s), method(s) to identify the presence and extent of statistical heterogeneity, and software package(s) used.	Page 4 and 5
	13e	Describe any methods used to explore possible causes of heterogeneity among study results (e.g. subgroup analysis, meta-regression).	Page 4 and 5
	13f	Describe any sensitivity analyses conducted to assess robustness of the synthesized results.	Page 4 and 5
Reporting bias assessment	14	Describe any methods used to assess risk of bias due to missing results in a synthesis (arising from reporting biases).	Page 4 and 5
Certainty assessment	15	Describe any methods used to assess certainty (or confidence) in the body of evidence for an outcome.	Page 4 and 5
**RESULTS**			

Section and Topic	Item #	Checklist item	Location where item is reported
Study selection	16a	Describe the results of the search and selection process, from the number of records identified in the search to the number of studies included in the review, ideally using a flow diagram.	Page 5
	16b	Cite studies that might appear to meet the inclusion criteria, but which were excluded, and explain why they were excluded.	Page 5
Study characteristics	17	Cite each included study and present its characteristics.	Page 5 and 6
Risk of bias in studies	18	Present assessments of risk of bias for each included study.	Page 7
Results of individual studies	19	For all outcomes, present, for each study: (a) summary statistics for each group (where appropriate) and (b) an effect estimate and its precision (e.g. confidence/credible interval), ideally using structured tables or plots.	Page 8 - 12
Results of syntheses	20a	For each synthesis, briefly summarise the characteristics and risk of bias among contributing studies.	Page 8 - 12
	20b	Present results of all statistical syntheses conducted. If meta-analysis was done, present for each the summary estimate and its precision (e.g. confidence/credible interval) and measures of statistical heterogeneity. If comparing groups, describe the direction of the effect.	Page 8 - 12
	20c	Present results of all investigations of possible causes of heterogeneity among study results.	Page 8 - 12
	20d	Present results of all sensitivity analyses conducted to assess the robustness of the synthesized results.	Page 12
Reporting biases	21	Present assessments of risk of bias due to missing results (arising from reporting biases) for each synthesis assessed.	NA
Certainty of evidence	22	Present assessments of certainty (or confidence) in the body of evidence for each outcome assessed.	Page 12 and 13
**DISCUSSION**			
Discussion	23a	Provide a general interpretation of the results in the context of other evidence.	Page 13
	23b	Discuss any limitations of the evidence included in the review.	Page 13 and 14

Section and Topic	Item #	Checklist item	Location where item is reported
	23c	Discuss any limitations of the review processes used.	Page 13 and 14
	23d	Discuss implications of the results for practice, policy, and future research.	Page 13 and 14
**OTHER INFORMATION**			
Registration and protocol	24a	Provide registration information for the review, including register name and registration number, or state that the review was not registered.	Page 3
	24b	Indicate where the review protocol can be accessed, or state that a protocol was not prepared.	Page 3
	24c	Describe and explain any amendments to information provided at registration or in the protocol.	NA
Support	25	Describe sources of financial or non-financial support for the review, and the role of the funders or sponsors in the review.	Page 14
Competing interests	26	Declare any competing interests of review authors.	Page 14
Availability of data, code and other materials	27	Report which of the following are publicly available and where they can be found: template data collection forms; data extracted from included studies; data used for all analyses; analytic code; any other materials used in the review.	Page 14

Table S2. PRISMA Abstract Checklist

Section and Topic	Item #	Checklist item	Reported (Yes/No)
**TITLE**			
Title	1	Identify the report as a systematic review.	Yes
**BACKGROUND**			
Objectives	2	Provide an explicit statement of the main objective(s) or question(s) the review addresses.	Yes
**METHODS**			
Eligibility criteria	3	Specify the inclusion and exclusion criteria for the review.	Yes
Information sources	4	Specify the information sources (e.g. databases, registers) used to identify studies and the date when each was last searched.	Yes
Risk of bias	5	Specify the methods used to assess risk of bias in the included studies.	Yes
Synthesis of results	6	Specify the methods used to present and synthesise results.	Yes
**RESULTS**			
Included studies	7	Give the total number of included studies and participants and summarise relevant characteristics of studies.	Yes
Synthesis of results	8	Present results for main outcomes, preferably indicating the number of included studies and participants for each. If meta-analysis was done, report the summary estimate and confidence/credible interval. If comparing groups, indicate the direction of the effect (i.e. which group is favoured).	Yes
**DISCUSSION**			
Limitations of evidence	9	Provide a brief summary of the limitations of the evidence included in the review (e.g. study risk of bias, inconsistency and	No

Section and Topic	Item #	Checklist item	Reported (Yes/No)
		imprecision).	
Interpretation	10	Provide a general interpretation of the results and important implications.	Yes
**OTHER**			
Funding	11	Specify the primary source of funding for the review.	No
Registration	12	Provide the register name and registration number.	Yes

Table S3. Key characteristics of the included studies

ID	ClinicalTrials.gov identifier	Study period	Study design	Country	Intervention	Cluster type	No.	Treatment and follow-up period
Jensen et al. (2025)	NCT04688775	December 2020 to October 2023	RCT	Belgium, Czech Republic, Denmark, Finland, France, Georgia, Germany, Greece, Italy, Japan, Netherlands, Norway, Portugal, Russia, Spain, Sweden, UK, and US	Eptinezumab 400mg/ placebo (intravenous infusion)	Episodic	231	4-week active treatment period, followed by a 12-week follow-up period, then an 8-week safety follow-up period
Tassorelli et al. (2025)	NCT05064397	September 2021 to June 2023	Single-arm open-	Denmark, Finland, France, Germany, Italy, Netherlands, Spain, the UK, and the USA	Eptinezumab 400mg (intravenous infusion	Chronic	131	48-week treatment period, followed by an 8-week

			label clinical trial		every 12 weeks)			safety follow-up period
Láinez et al (2021)	NCT02438826	July 2015 to June 2019	Single-arm open-label clinical trial	Belgium, Canada, Denmark, Finland, France, Germany, Greece, Italy, Netherlands, Spain, United Kingdom, and United States	Galcanezumab 300 mg, administered subcutaneously once monthly	chronic	233	12-week double-blind treatment period and an optional 1-year open-label treatment period, followed by a 16-week safety follow-up period
Mecklenburg et al. (2025)	NCT04970355	December 2021 to September 2023	RCT	United States, Spain, Germany, and the United Kingdom	Erenumab 70 mg/ placebo administered subcutaneously once a month	Chronic	81	6-week treatment period, followed by a 4-week safety follow-up period
Goadsby et al. (2019)	NCT02397473	May 2015 to June 2018	RCT	35 sites in Europe and North America.	Galcanezumab (at a dose of 300 mg)/ placebo, both of which were administered	episodic	106	8-week treatment period, followed by a 4-month follow-up period

					subcutaneously at baseline and at 1 month			
Dodick et al. (2020)	NCT02438826	July 2015 to March 2018	RCT	Belgium, Canada, Denmark, Finland, France, Germany, Greece, Italy, Netherlands, Spain, United Kingdom, and United States	Galcanezumab (at a dose of 300 mg/ placebo, both groups received three injections at each dosing visit	chronic	237	12-week double-blind treatment period

RCT: randomized controlled trial, No.: number of participants

Table S4. Risk of bias summary for non-randomized studies (ROBINS-I V2) tool for single-arm trials

**Study**	**Bias due to confounding***	**Bias in selection of participants**	**Bias in classification of interventions**	**Bias due to missing data**	**Bias arising from measurement of the outcome**	**Bias in selection of the reported result**	**Overall Risk of bias judgment**
**Galcanezumab**	**Moderate**	**Moderate**	**Low**	**Moderate**	**Moderate**	**Low**	**Moderate**
**Eptinezumab**	**Moderate**	**Low**	**Low**	**Moderate**	**Moderate**	**Low**	**Moderate**

*** All non-randomized studies are subject to bias due to confounding factors.**

Table S5. Subgroup analyses for efficacy outcomes

Subgroup		change from baseline in mean number of weekly attacks				50% or greater responders				30% or greater responders				PGIC rating of "much improved" or "very much improved "			
		No.	ES (95% CI)	P	I^2	No.	ES (95% CI)	P	I^2	No.	ES (95% CI)	P	I^2	No.	ES (95% CI)	P	I^2
Study design	RCT	4	-7.79 (-10.97- -4.61)	P < 0.0001	94.90%	4	0.51 (0.26-0.76)	P < 0.0001	94.90%	4	0.63 (0.47-0.78)	p < 0.0001	87.2%,	3	0.42 (0.15-0.73)	p < 0.0001	94.4%,
	Single-arm clinical trial	1	-4.98 (-7.29- -2.67)	N/A	N/A	1	0.31 (0.24-0.40)	N/A	N/A	1	0.43 (0.35-0.52)	N/A	N/A	1	0.41 (0.32-0.51)	N/A	N/A
Medication	Eptinezumab	2	-6.70 (-10.02- -3.38)	0.0364	77.20%	2	0.49 (0.17-0.82)	P < 0.0001	96.5%,	2	0.59 (0.29-0.85)	p < 0.0001	95.4%,	1	0.41 (0.32-0.51)	N/A	N/A
	Galcanezumab	2	-7.81 (-14.96- -0.65)	P < 0.0001	95.8%,	2	0.52 (0.08-0.94)	P < 0.0001	97.30%	2	0.60 (0.30-0.87)	0.0003	92.5%,	2	0.46 (0.05-0.90)	p < 0.0001	97.2%,
	Erenumab	1	-7.30 (-9.93- -4.67)	N/A	N/A	1	0.32 (0.18-0.48)	N/A	N/A	1	0.59 (0.42-0.74)	N/A	N/A	1	0.37 (0.22-0.53)	N/A	N/A
Cluster	Episodic	2	-	0.05	72.70	2	0.71	0.17	45.40	2	0.74	0.82	0.00	1	0.71	N/A	N/A

type			9.90 (-12.97- -6.84)	55	%		(O.60-0.81)	6	%		(0.67-0.81)	2	%		(0.57-0.83)		
	Chronic	3	-7.23 (-9.86--4.60)	P < 0.0001	85.8 %,	3	0.30 (0.25-0.35)	0.7423	0.00 %	3	0.47 (0.40-0.54)	0.2333	31.30 %	3	0.33 (0.20-0.47)	0.007	79.80 %

PGIC: Patient Global Impression of Change.

Table S6. GRADE summary of findings for randomized controlled trials (proportional outcomes)

Outcome	No. of participants	Pooled estimate (95 % CI)	Risk of bias	Inconsistency	Indirectness	Imprecision	Publication bias	Certainty of evidence
Any TEAEs	202	0.44 (0.21–0.68)	Low	Downgrade	No indirectness	No imprecision	Unlikely	⊕⊕⊕⊖ Moderate
SAEs	202	0.01 (0.00–0.05)	Low	No inconsistency	No indirectness	Downgrade	Unlikely	⊕⊕⊖⊖ Low
Discontinuations due to AEs	202	0.02 (0.00–0.04)	Low	No inconsistency	No indirectness	Downgrade	Unlikely	⊕⊕⊖⊖ Low
Change from baseline in weekly attacks	311	-7.79 (-10.97 to -4.61)	Low	Downgrade	No indirectness	No imprecision	Unlikely	⊕⊕⊕⊖ Moderate
≥ 30 % responder rate	309	0.63 (0.47–0.78)	Low	Downgrade	No indirectness	No imprecision	Unlikely	⊕⊕⊕⊖ Moderat

								e
≥ 50 % responder rate	309	0.51 (0.26–0.76)	Low	Downgrade	No indirectness	No imprecision	Unlikely	⊕⊕⊕⊖ Moderate
PGIC (“much / very much improved”)	192	0.42 (0.15–0.73)	Low	Downgrade	No indirectness	Downgrade	Unlikely	⊕⊕⊖⊖ Low

TEAEs: Treatment-Emergent Adverse Events, SAEs: Serious Adverse Events, AEs: Adverse Events, PGIC: Patient Global Impression of Change.

Figure S1. Subgroup Analysis of Safety Outcomes by Study Design; (A) Participants with ≥1 TEAE, (B) Participants with ≥1 SAE, (C) Participants with ≥1 AE leading to withdrawal

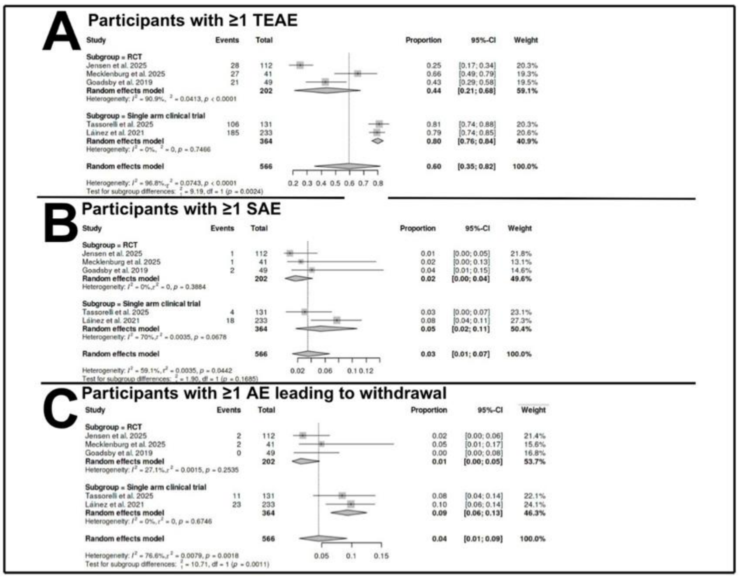

TEAEs: Treatment-Emergent Adverse Events, SAEs: Serious Adverse Events, AEs: Adverse Events.

Figure S2. Subgroup Analysis of Safety Outcomes by Medication Type;(A) Participants with ≥1 TEAE, (B) Participants with ≥1 SAE, (C) Participants with ≥1 AE leading to withdrawal

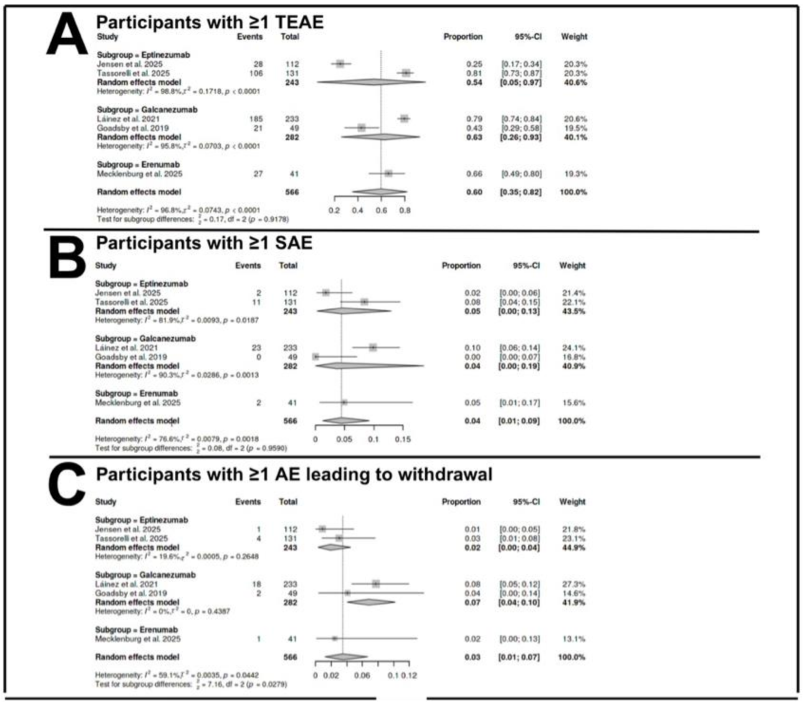

TEAEs: Treatment-Emergent Adverse Events, SAEs: Serious Adverse Events, AEs: Adverse Events.

Figure S3. Subgroup analysis of efficacy outcomes by study design; (A) change from baseline in number of weekly Attacks, (B) 50% or greater responders, (C) 30% or greater responders, (D) Patient-reported Patient Global Impression of Change rating of “much improved” or “very much improved”

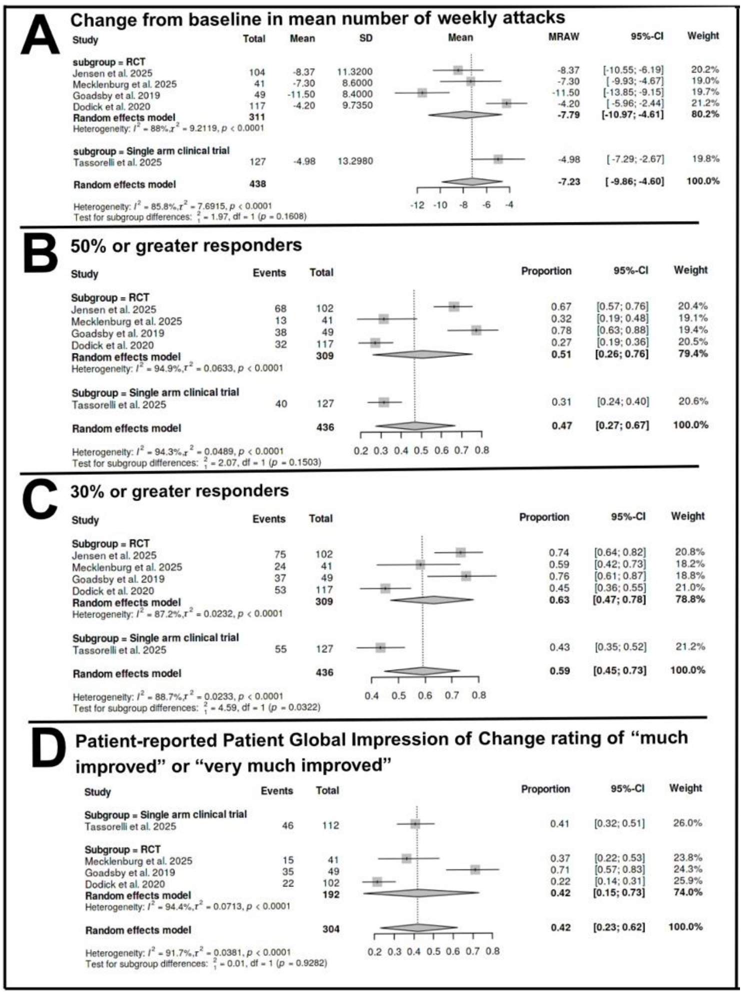

Figure S4. Subgroup analysis of efficacy outcomes by medication type. (A) change from baseline in number of weekly Attacks, (B) 50% or greater responders, (C) 30% or greater responders, (D) Patient-reported Patient Global Impression of Change rating of "much improved" or "very much improved"

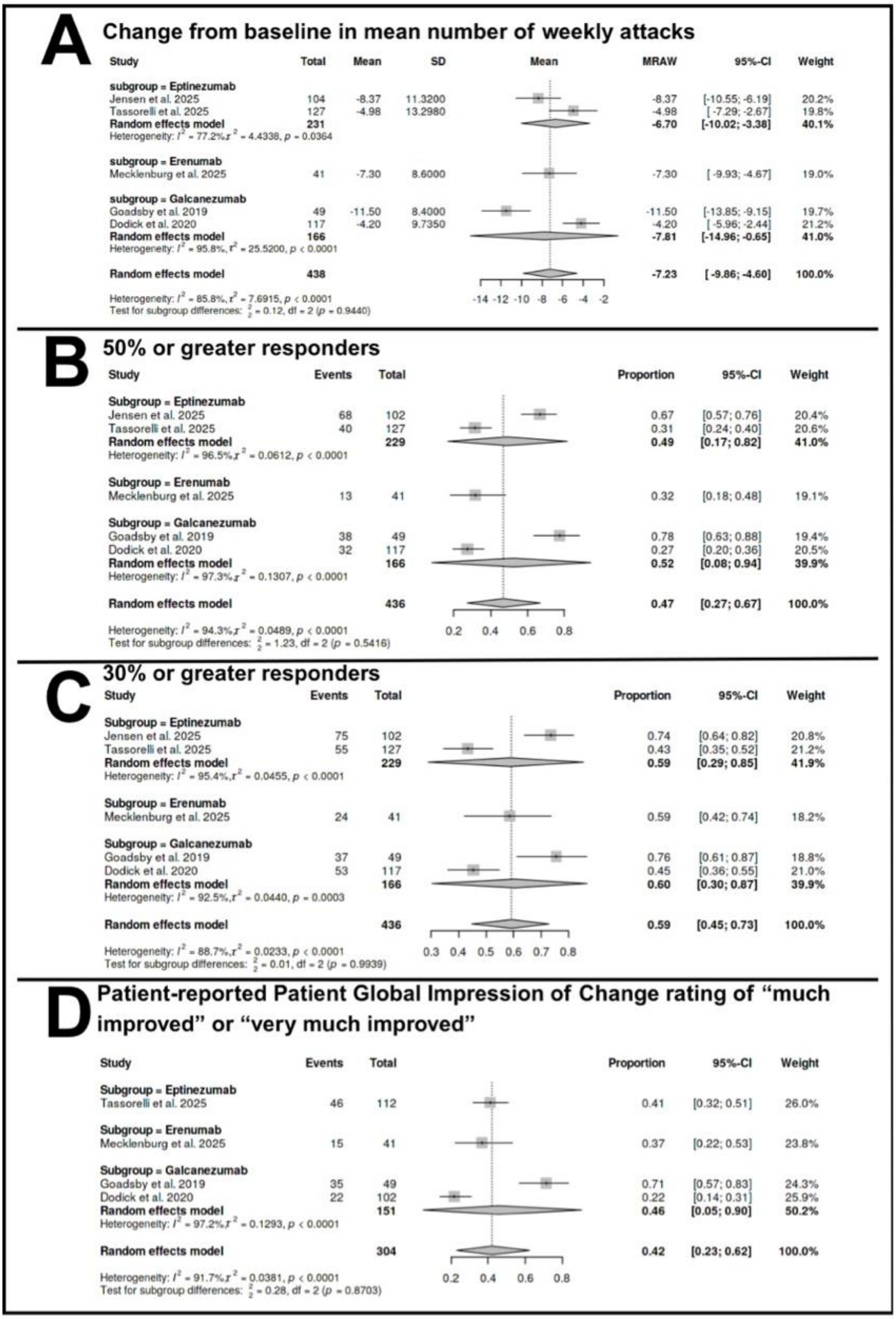

Figure S5. Subgroup analysis of efficacy outcomes Cluster headache type (Episodic vs Chronic); (A) change from baseline in number of weekly Attacks, (B) 50% or greater responders, (C) 30% or greater responders, (D) Patient-reported Patient Global Impression of Change rating of "much improved" or "very much improved"

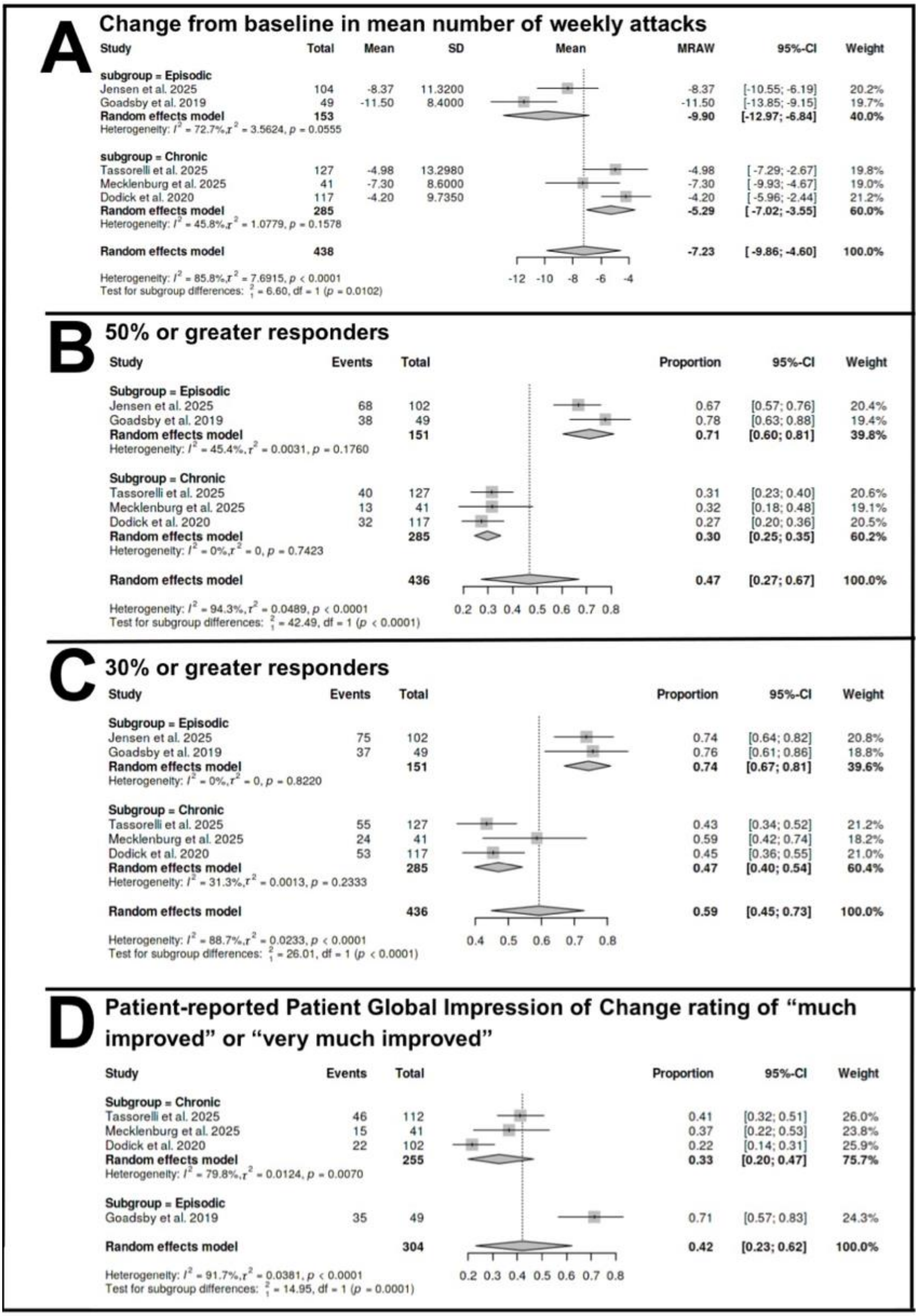

Figure S6. Funnel Plots for Publication Bias for the outcomes: (A) Participants with ≥1 TEAE, (B) 50% or greater responders, (C) 30% or greater responders

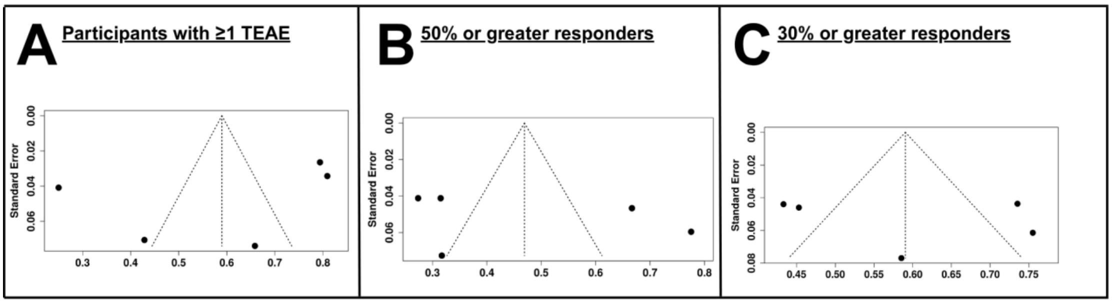

TEAEs: Treatment-Emergent Adverse Events, SAEs.

Figure S7. Leave-One-Out Sensitivity Analysis for Safety Outcomes; (A) Participants with ≥1 TEAE, (B) Participants with ≥1 SAE, (C)Participants with ≥1 AE leading to withdrawal

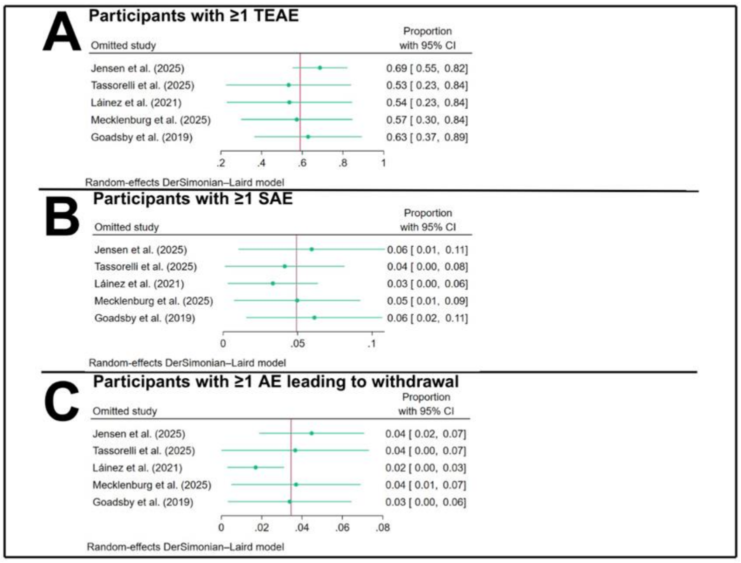

TEAEs: Treatment-Emergent Adverse Events, SAEs: Serious Adverse Events, AEs: Adverse Events.

Figure S8. Leave-One-Out Sensitivity Analysis for Efficacy Outcomes; (A) 50% or greater responders, (B) 30% or greater responders, (C) Patient-reported Patient Global Impression of Change rating of "much improved" or "very much improved"

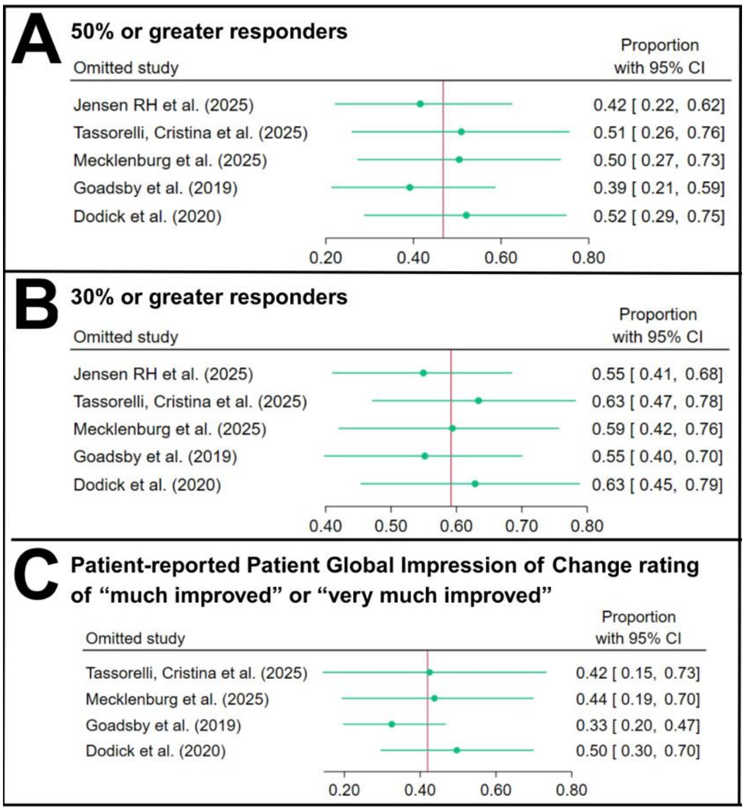

Supplement: Supplementary file 1 — Supplementary Material 1. [file 12883_2026_4733_MOESM1_ESM.pdf]
